# Portal dosimetry scripting application programming interface (PDSAPI) for Winston‐Lutz test employing ceramic balls

**DOI:** 10.1002/acm2.13043

**Published:** 2020-10-24

**Authors:** Yao Hao, Matthew C. Schmidt, Yu Wu, Nels C. Knutson

**Affiliations:** ^1^ Department of Radiation Oncology Washington University School of Medicine St. Louis MO USA

**Keywords:** BB, displacement, DoseLab, PDSAPI, RIT, shift, Winston‐Lutz

## Abstract

**Purpose:**

Stereotactic radiosurgery (SRS) and stereotactic body radiation therapy (SBRT) treatments require a high degree of accuracy. Mechanical, imaging, and radiation isocenter coincidence is especially important. As a common method, the Winston‐Lutz (WL) test plays an important role. However, weekly or daily WL test can be very time consuming. We developed novel methods using Portal Dosimetry Scripting Application Programming Interface (PDSAPI) to facilitate the test as well as documentation.

**Methods:**

Winston‐Lutz PDSAPI was developed and tested on our routine weekly WL imaging. The results were compared against two commercially available software RIT (Radiological Imaging Technology, Colorado Springs, CO) and DoseLab (Varian Medical Systems, Inc. Palo Alto, CA). Two manual methods that served as ground truth were used to verify PDSAPI results. Twenty WL test image data sets (10 fields per tests, and 200 images in total) were analyzed by these five methods in this report.

**Results:**

More than 99.5% of WL PDSAPI 1D shifts agreed with each of four other methods within ±0.33 mm, which is roughly the pixel width of a‐Si 1200 portal imager when source to imager distance (SID) is at 100 cm. 1D shifts agreement for ±0.22 mm and 0.11 mm were 96% and 63%, respectively. Same trend was observed for 2D displacement.

**Conclusions:**

Winston‐Lutz PDSAPI delivers similar accuracy as two commercial applications for WL test. This new application can save time spent transferring data and has the potential to implement daily WL test with reasonable test time. It also provides the data storage capability, and enables easy access to imaging and shift data.

## 1 INTRODUCTION

Stereotactic radiosurgery (SRS) is a noninvasive and a highly precise form of radiation therapy. It was originally developed to treat small brain lesion or functional abnormalities of the brain. When radiosurgical techniques are used to treat extracranial diseases, it is referred to as stereotactic body radiotherapy (SBRT).^1^ Both SRS and SBRT deliver highly concentrated doses per fraction with steep dose fall‐off and high conformality to spare organs at risk. The corresponding quality control procedures prior radiation treatment are essential to assure delivered accuracy and precision. One of the important factors is the coincidence of imaging, mechanical, and radiation isocenters.

Lutz, Winston, and Maleki^2^ developed a method to verify the alignment using a film prior to each SRS treatment. This quality assurance (QA) procedure, Winston‐Lutz (WL) test, was originally designed to verify the coincidence of radiation isocenter and mechanical isocenter in a 6MV linear accelerator‐based SRS system. With the advent of on‐board imagers, WL test has been modified by using electronic portal imaging device (EPID). Electronic portal imaging can be very useful due to its nature of fast image acquisition, digital format, high spatial resolution, as well as potential Portal Dosimetry.^3^

Several American Association of Physicists in Medicine (AAPM) task group (TG) reports recommend verifying congruence between radiation and imaging isocenters.^4^, ^5^, ^6^, ^7^, ^8^, ^9^ Per TG142, the daily accuracy of imaging and treatment coordinate coincidence should be within 1 mm for SBRT techniques.^6^ TG179 recommends that the geometric calibration, the relationship between imaging and LINAC radiation isocenters, should be tested daily following the procedure described in AAPM TG‐66.^5^, ^8^ While the recommended test only incorporates four gantry cardinal angles to compare the ball bearing (BB) image centroid to field delimiting apertures. More combinations of gantry, couch, and collimator are beneficial to delineate the performance of corresponding axis of motion within the system. For SRS, daily WL test is a more comprehensive test to verify the accuracy of the laser, mechanical isocenter shift over time, couch movement accuracy, and multileaf collimator (MLC) position accuracy.

The Eclipse Scripting Application Programming Interface (ESAPI) was introduced into the Eclipse Treatment Planning System (Varian, Palo Alto, CA) in 2012 to provide access to treatment planning and segmentation data. In 2013, Portal Dosimetry Scripting Application Programming Interface (PDSAPI), a programming interface and a software library for Portal Dosimetry (Varian, Palo Alto, CA), was developed to access to the dosimetric images of modulated fields. Users can write custom software applications to access the data model from Portal Dosimetry. This can be done either by integrating scripts into the Portal Dosimetry user interface, or run the script as stand‐alone executables.^10^, ^11^

Generally, WL is done by using a film or portal imager. These films or portal images can be analyzed by commercial software. However, either scanning films or transferring DICOM images is time consuming, especially when considering a daily implementation of the WL test. Also, current commercial software solutions do not have the capability to additionally perform ongoing QA for external patient surface tracking systems, and cone‐beam computed tomography (CBCT) imaging system measured shifts as well as isocenter drifts over time.

Many algorithms for WL portal image analysis have employed edge detection and center of mass calculations.^12^, ^13^, ^14^ However, the accuracy can be suboptimal for low‐resolution images. Winey et al developed a fast WL algorithm, using a double convolution, to find cone and sphere centers separately.^15^ They provided a robust solution for low‐resolution images. However, these algorithms require the extraction of data into a third party software as opposed to direct analysis within the oncology information system.

The goals of this study are to provide an integrated solution to speed up the isocenter congruence QA on a phantom with ceramic balls by using a Treatment Planning System (TPS) Application Programming Interface (API) to analyze images online, and provide the functions of recording and reporting in a commercial oncology information system. Besides routine WL test, the application also allows the user to test surface monitoring system.

A custom software application was developed to enable the delivery of a daily WL with a reasonable time frame, help user to quantify isocenter congruence, and provide documentation tool for isocenter drifts over time.

## 2 METHODS

**Fig. 1:**
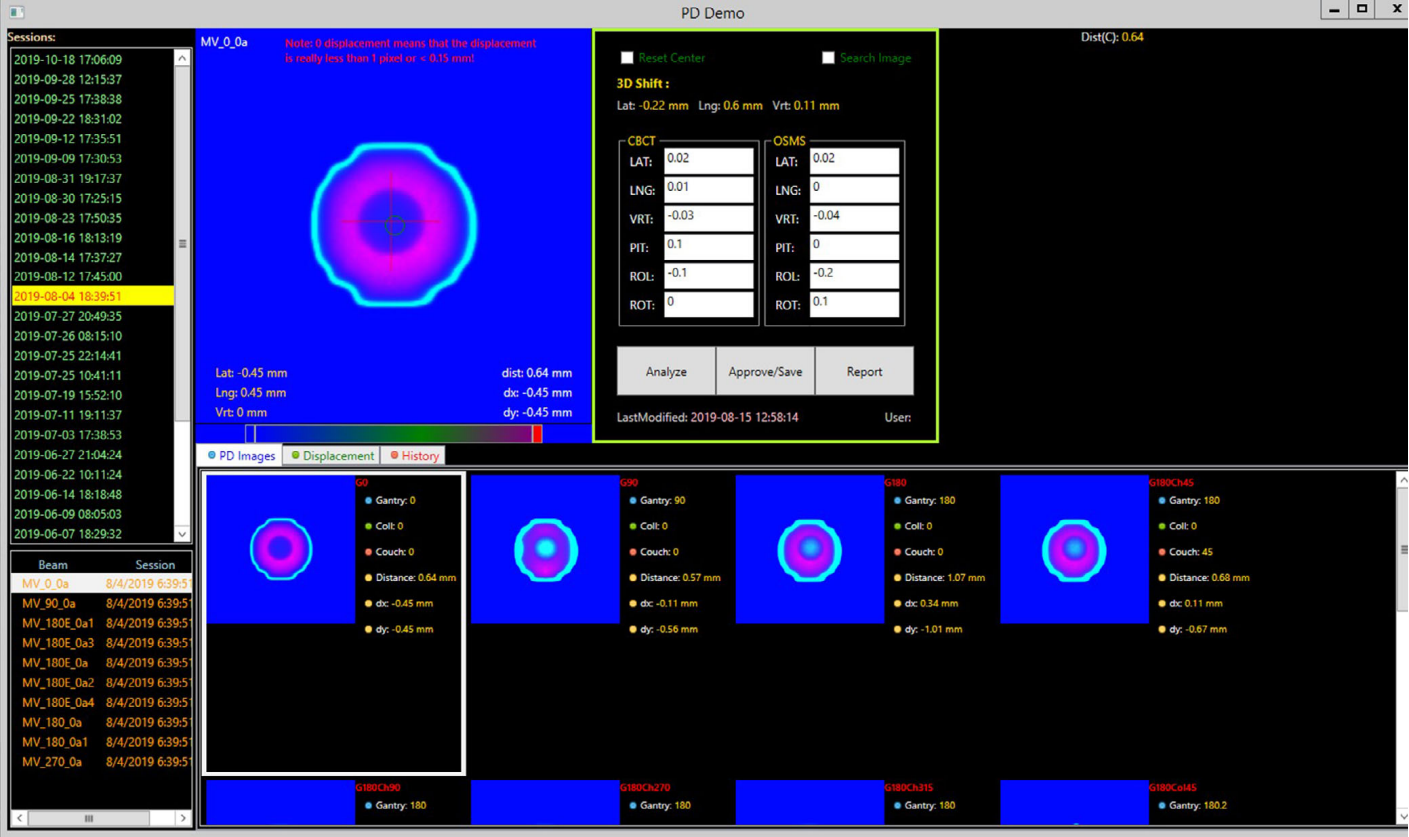
Winston‐Lutz PDSAPI interface.

A new PDSAPI application for the analysis of WL tests was developed. This application’s feature set includes loading in images from the Portal Dosimetry application, allowing the user to determine the results of the WL test through either algorithmic or manual analysis; the user then inputs shift data from daily imaging QA and Optical Surface Monitoring System (OSMS, Varian, CA). Finally, the application reports shift data as Portable Document Format, and allows for physics review of QA results from database. The WL PDSAPI can be launched as a script from the Portal Dosimetry workspace. The interface is shown below in Fig. 1.

**Fig. 2:**
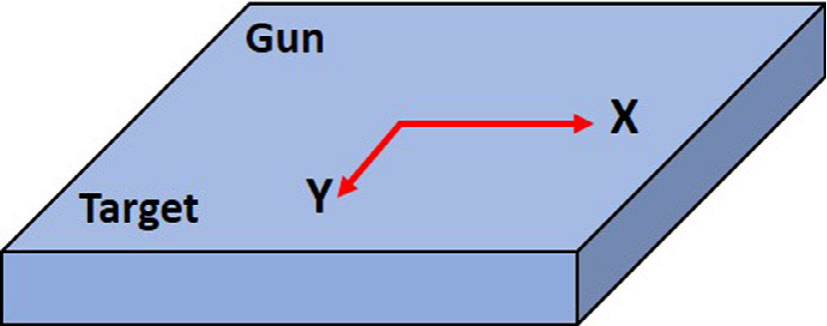
Portal imager coordinate system.

Once images are loaded, WL PDSAPI uses AForge image processing to search boundaries of radiation field and ball, and further finds BB center (x_B,_ y_B_) and field center (x_F,_ y_F_). Ball center horizontal shift (H_B_) and vertical shift (V_B_) relative to field center were calculated correspondingly. Fig. 2 shows the coordinate system of EPID of this application. X and Y represent the horizontal and vertical direction of the imager, respectively.

Equations built into the application are:

(1) $$H_{B}=x_{B}‐x_{F}$$

(2) $$V_{B}=y_{B}‐y_{F}$$

(3) $$R=\sqrt{H_{B}^{2}+V_{B}^{2}}$$

The AForge.NET image processing library utilizes a custom implementation of the *connected components labeling* algorithm to categorize regions within the image based on pixel intensity value.^16^, ^17^ The outlines of these categorized regions, denoted “blobs” by AForge, are analyzed by calculating the mean points between the outline and verifying the differences between an assumed shape (commonly a circle or a rectangle). In the current implementation of shape determination, the outline of the field and ball is reorganized into a circle. If a circle is undetectable, the field and ball center points are assumed to be the geometrical center of the corresponding blobs.

Portal imager coordinate system in this study is identical to RIT software (Radiological Imaging Technology, Colorado Springs, CO). Horizontal direction of DoseLab (Varian Medical Systems, Inc. Palo Alto, CA) is same as RIT, while the vertical direction is at the opposite. Therefore, the vertical coordinate shifts were manually reversed with the vertical shift results from DoseLab to match other analysis methods.

**Fig. 3:**
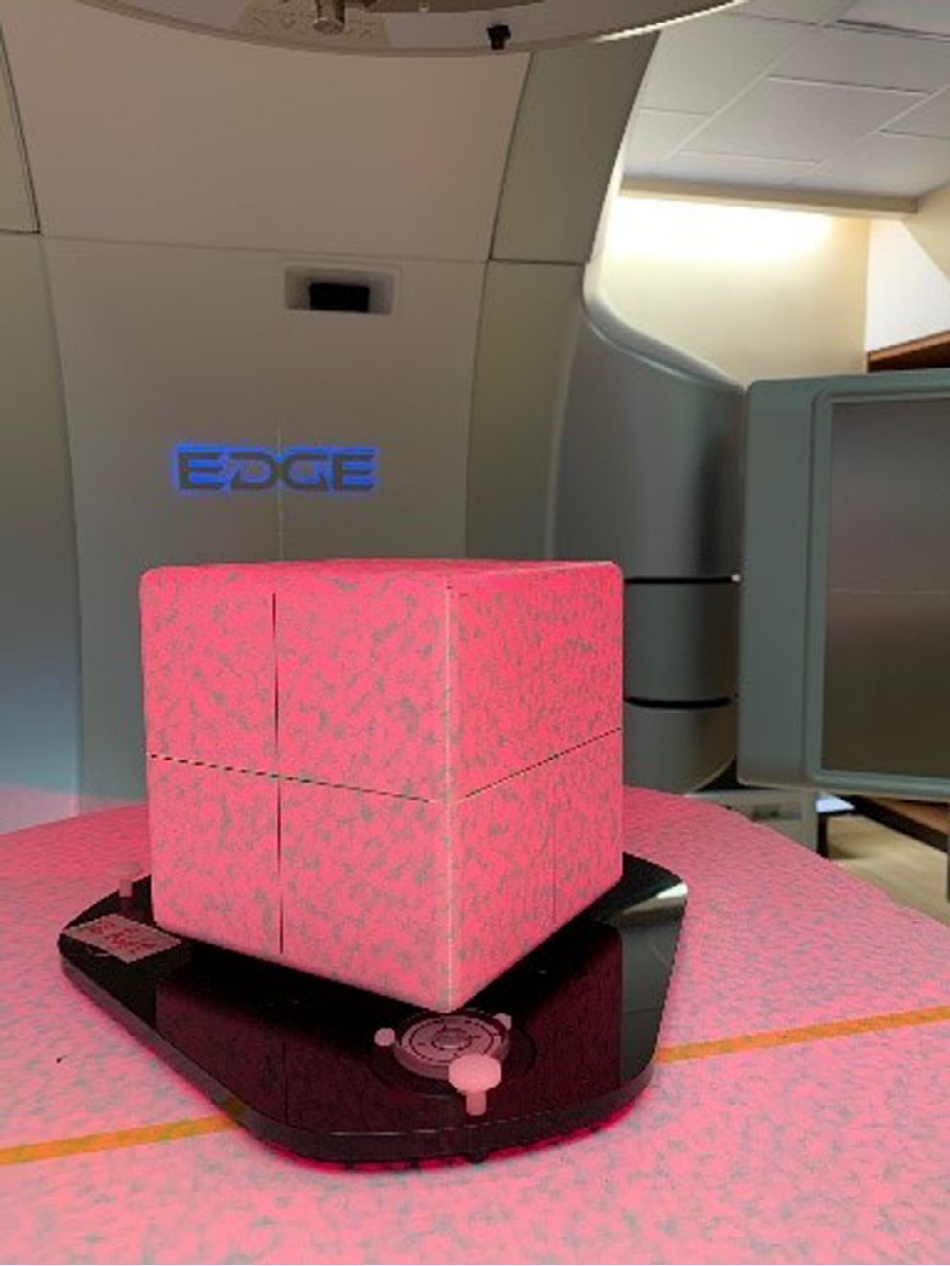
OSMS Isocube phantom.

In this study, the field was shaped by Varian High‐Definition 120 multi‐leaf collimator with jaw setting 2 cm × 2 cm. OSMS Isocube phantom was scanned and planned in Eclipse™ TPS (Varian Medical Systems, Palo Alto, CA, USA). The record and verify system used in this study is ARIA 13.7 (Varian Medical Systems, Palo Alto, CA, USA). Phantom is composed of five embedded alumina ceramic BBs arranged at different locations in a plastic cube as shown in Fig. 3. The central BB in the phantom was targeted for this test. The ball size is 7.5 mm in diameter. MLCs are snapped around it with 5 mm margin. The LINAC model used is Varian TrueBeam EDGE with a‐Si 1200 portal imager (Varian Medical Systems, Palo Alto, CA, USA). Ten field combinations, as shown in Table 1, were used for the test. All fields have the same monitor unit (MU). To speed up the delivery, 6MV flattening filter free (FFF) with high dose rate was used.

**Table 1:** WL test machine parameters.

Gantry (Degree)	Collimator (Degree)	Couch (Degree)
0	0	0
90	0	0
180	0	0
180	0	45
180	0	90
180	0	270
180	0	315
180	45	0
180	315	0
270	0	0

To begin the QA procedure, the OSMS phantom was placed at the end of the couch with six degrees of freedom (6DoF) and aligned using OSMS system. Pitch, roll, and rotation readings from OSMS were all within 0.2 degree, and vertical, longitudinal, and lateral readings were all within 0.2 mm. Subsequently, a CBCT was acquired, and registered to the computed tomography (CT) scan of the phantom. CBCT shifts were recorded in the WL PDSAPI, as well as the post‐shift OSMS readings. KV‐pairs were acquired afterwards to verify the CBCT shifts.

Portal images were then acquired for all 10 fields and exported for analysis using two commercially available software products, RIT and DoseLab. Two manual methods served as a benchmark to verify PDSAPI results. Twenty WL test image data sets (200 images in total) were analyzed by these five methods in this study.

The first manual comparison methods are a direct distance measuring method. This method uses Eclipse version 13.7 Portal Dosimetry application. Image intensity was set to obtain a consistent level for acceptable visualization of both ball edge and field edge. The digital graticule was used as reference to find ball center shift from field center. Four distances were measured to determine field center, and another four used to find ball center. Those eight measurements include vertical and horizontal distance from the center of digital graticule to ball boundary, vertical and horizontal distance from the center of digital graticule to field boundary, and vertical and horizontal width of both ball and field. All measurements were performed along with digital graticule X and Y axes. Measurements for ball and field will decide the centers for ball and field separately. By comparing those two centers, vertical and horizontal shifts were determined with the following equations.

(4) $$H_{B}={(x}_{B,CR}‐\frac{x_{B,LR}}{2})‐{(x}_{F,CR}‐\frac{x_{F,LR}}{2})$$

(5) $$V_{B}={(y}_{B,CA}‐\frac{y_{B,AP}}{2})‐{(y}_{F,CA}‐\frac{y_{F,AP}}{2})$$

where,

B represents BB, and F represents field.

$x_{B,CR}$ is the distance from the center of digital graticule to the right side of ball boundary,

$x_{B,LR}$ is the distance from the left side of ball boundary to the right side of it,

$y_{B,CA}$ is the distance from the center of digital graticule to the anterior side of ball boundary,

$y_{B,AP}$ is the distance from the posterior side of the ball boundary to the anterior of it,

Same definition applied to the field measurements: $x_{F,CR}$, $x_{F,LR}$, $y_{F,CA}$, $y_{F,AP}$.

For the field with nonzero collimator angle, the image was rotated in the Portal Dosimetry application as same as collimation angle. Then, the above measurements were performed.

The second manual method is pixel values‐based measuring method. This method also utilized Eclipse version 13.7 Portal Dosimetry application. Image intensity was set consistently with the first method. The Area Histogram tool allows the user to fit the region of interest (ROI) into a rectangular region, and also can provide pixel position of the upper left corner (x_UL_, y_UL_) of ROI and size (x_S_, y_S_) of ROI. Rectangular region can be set as close as possible to the outer border of the field or ball. Based on the ROI parameters for both field and ball, one can decide the coordinates of their center, respectively, relative to panel center (x_C_, y_C_). The shifts can be decided by the following equations:

(6) $$H_{B}=\left(\frac{x_{B,UL}‐x_{B,S}}{2}‐x_{C}\right)\times PSI‐\left(\frac{x_{F,UL}‐x_{F,S}}{2}‐x_{C}\right)\times PSI$$

(7) $$V_{B}=\left(\frac{y_{B,UL}‐y_{B,S}}{2}‐y_{C}\right)\times PSI‐\left(\frac{y_{F,UL}‐y_{F,S}}{2}‐y_{C}\right)\times PSI$$

(8) $$where\;PSI\;=\;\left(\frac{\mathrm{SID}}{100}\right)\;\times \;Pixel\;Resolution$$

Here, B represents BB and F represents field. Pixel size at isocenter (PSI) is the scaling factor, and PSI equals 1 at source to imager distance (SID) at 100 cm. PSI depends on the vertical position of the EPID imager during the image acquisition.

PDSAPI‐resolved field size and BB size (diameter) for all 20 image sets were also investigated. Due to the remarkable difference between in and out of field, the field size variation is much less than BB size. However, the matched fields are only used to find their centers. The size itself can be changed by the threshold applied, phantom setup, imager response, as well as machine performance. OSMS Isocube has four BBs around the central BB. For some gantry, collimator, and couch setting combinations, portion of other BB can go inside the field. This will affect the matched BB and field circles. Threshold was applied to make field and BB more distinguishable, so the matched field and BB sizes are smaller than their physical sizes.

PDSAPI accuracy was also evaluated for all our clinically used energies. The sequence of the fields follows the same order as Table 1. Four plans were made with different energies. Once phantom was aligned, four plans were delivered one after another without any setup change.

Comparison of contrast to noise ratio (CNR) at various MU levels was performed for both WL phantoms. Histogram tool in Portal Dosimetry was used. ROI was selected as a square with dimension as 15 by 15 pixels. CNR was defined as the ratio of absolute mean pixel value difference between inside BB and field (outside BB) and field noise.

## 3 RESULTS

**Fig. 4:**
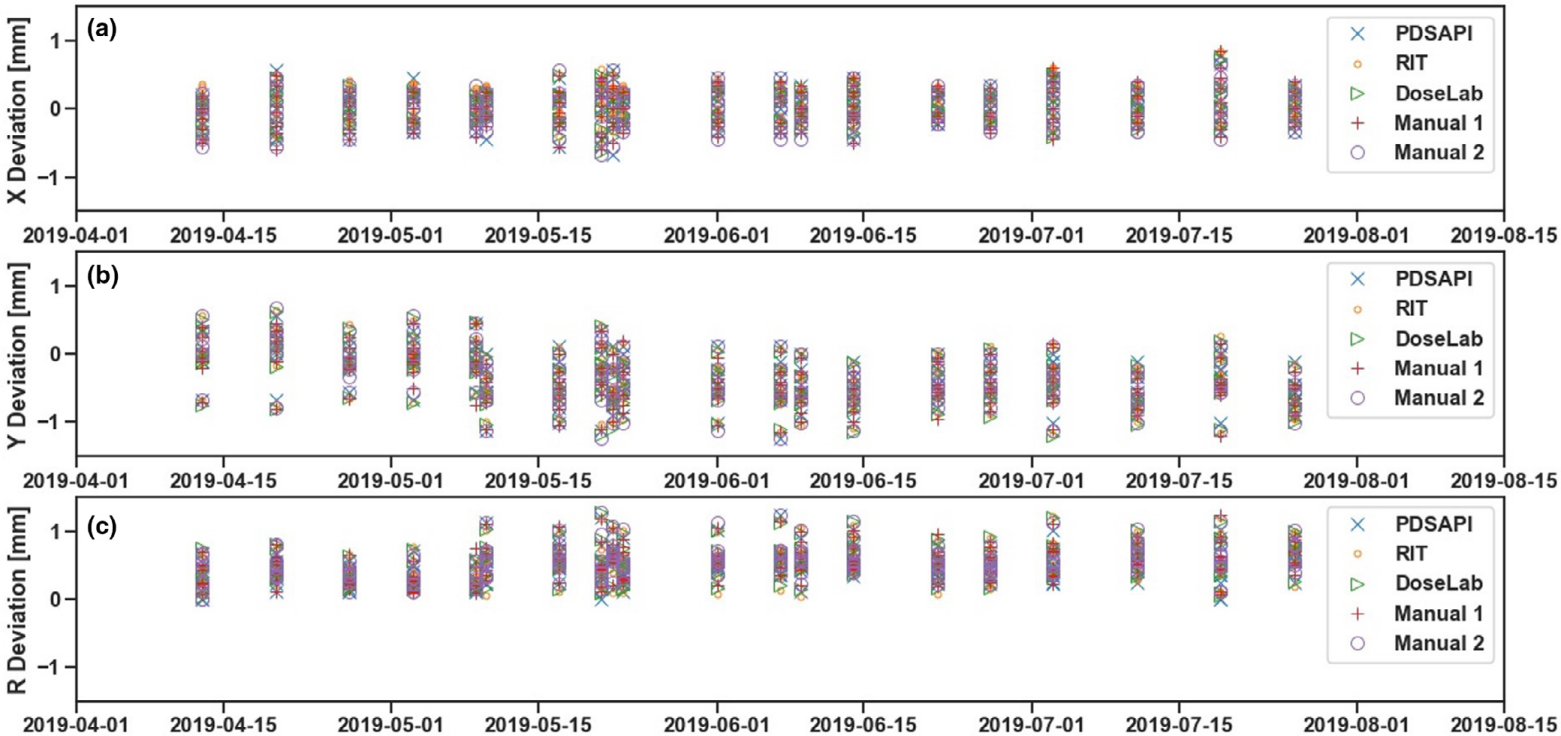
Horizontal, vertical, and 2D deviations of all five methods.

The shift results of all five methods are shown below in Fig. 4. X deviation is the horizontal shift, Y deviation is the vertical shift, and R is the 2D displacement. WL PDSAPI results were compared against all the other four methods. Manual 1 represents direct distance measuring method. Manual 2 represents Histogram tool measuring method.

**Fig. 5:**
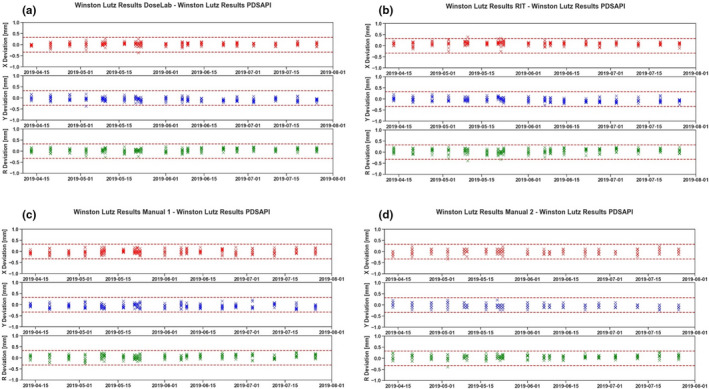
PDSAPI deviation comparisons against the rest of the methods. (a) Deviation of DoseLab and PDSAPI, (b) Deviation of RIT and PDSAPI, (c) Deviation of manual methods 1 and PDSAPI, (d) Deviation of manual methods 2 and PDSAPI.

There are 1600 pairs of 1D comparisons in total. Figures below (Fig. 5a‐d) show the differences between PDSAPI and all other four methods. Red dash lines represent ±0.33 mm away from no difference. About 400 observation pairs for each method are compared against PDSAPI—200 are horizontal shifts, and 200 are vertical shifts. Overall, about 99.7% (1595 pairs) of the differences are within ±0.33 mm (about 1 pixel width), 97.6% (1563 pairs) of them are within ±0.22 mm (about 2/3 pixel width), and 61.9% (1159 pairs) of them are within ±0.11 mm (about 1/3 pixel width). For PDSAPI 2D displacement (R) comparisons, the percentage falling into each threshold is very similar. They are 67.6%, 96.5%, and 99.8% for the range of ±0.11 mm, ±0.22 mm, and ±0.33 mm, correspondingly.

Total number of 2D comparison is 800 observation pairs. All 2D differences are within ±0.33 mm for DoseLab and manual 1 methods. Only one pair (0.5%) 2D difference are more than ±0.33 mm for RIT and manual 2 methods each.

**Fig. 6:**
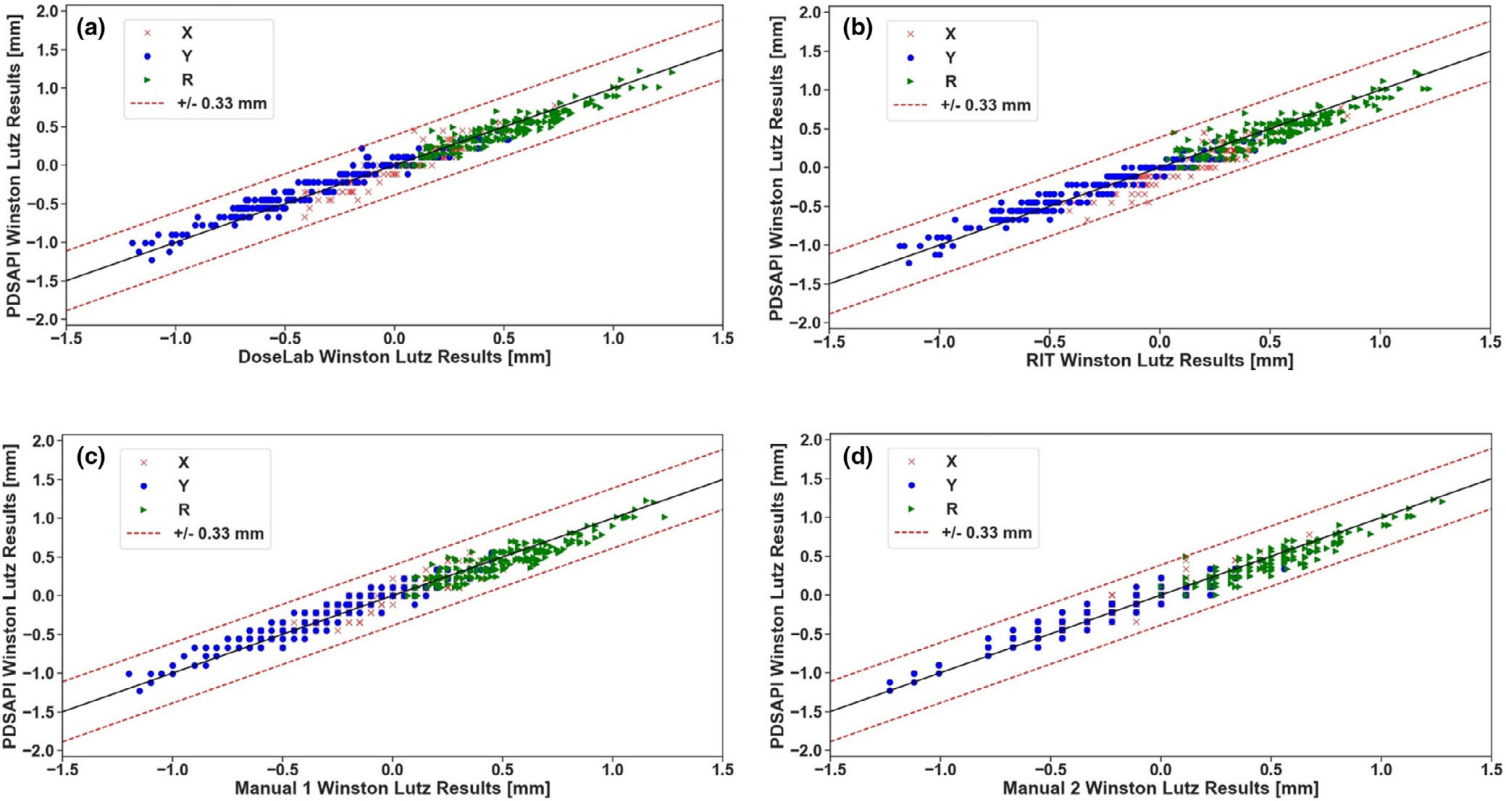
PDSAPI correlation with rest of the methods. (a) Correlation of DoseLab and PDSAPI, (b) Correlation of RIT and PDSAPI, (c) Correlation of manual methods 1 and PDSAPI, (d) Correlation of manual methods 2 and PDSAPI.

The correlations between PDSAPI and rest of the methods were also investigated as shown in Fig. 6a‐d. Red dash lines represent ±0.33 mm away from perfect positive correlation. All of the points fall within the range of 1 pixel ROI (±0.33 mm).

**Fig. 7:**
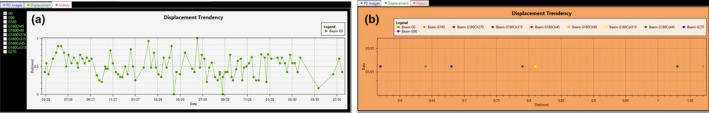
Displacement trend views. (a) Displacement by beam, (b) Displacement by image data set.

As shown in Fig. 7a,b, additional features of the PDSAPI application allow the user to review the displacement trends for single or multiple field(s) under the option “History”, or to review displacement for any WL image data sets under the option “Displacement”.

**Fig. 8:**
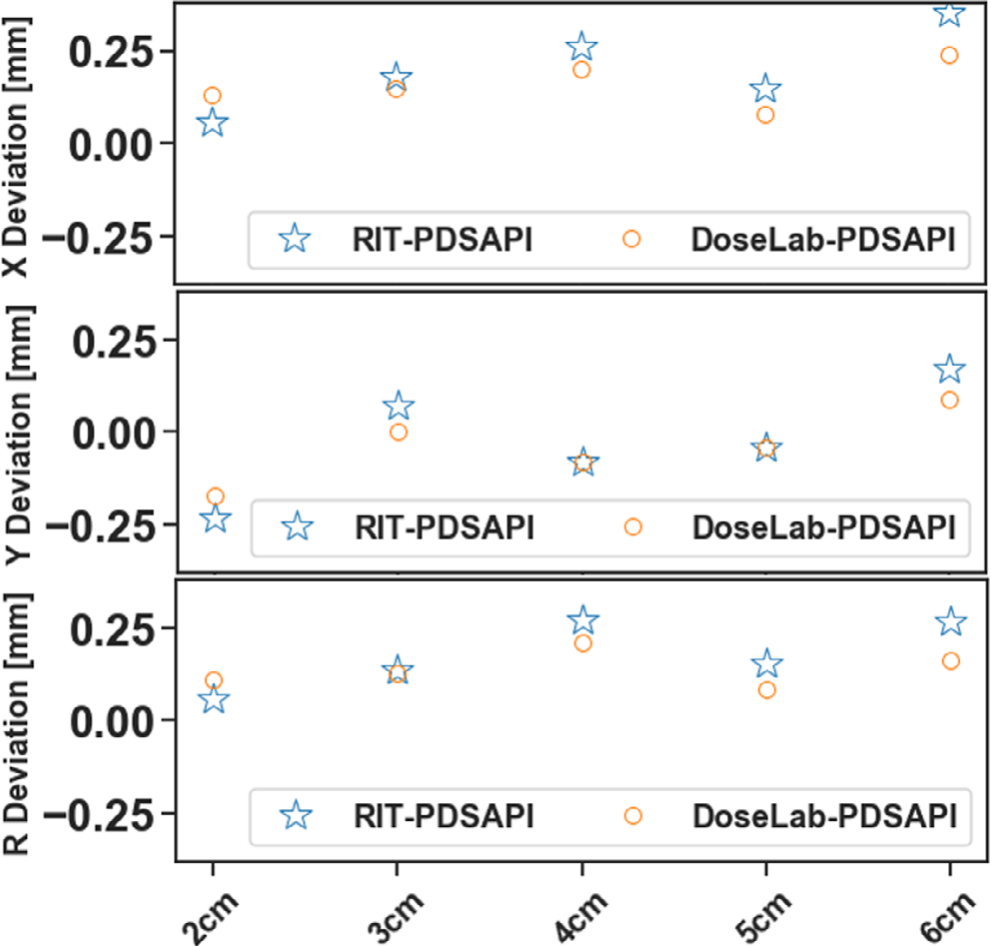
PDSAPI accuracy vs field size.

Fig. 8 shows the PDSAPI results comparing to two commercial software for different field sizes with fixed gantry angle. Phantom was kept at the same position. The only changes between fields are Jaw and MLC shapes. No significant difference on the accuracy of PDSAPI was observed.

**Fig. 9:**
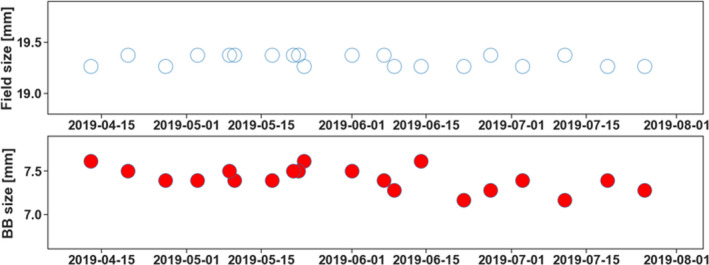
Field size and BB size congruence.

Variation of PDSAPI decided field size and BB size were also studied. Figure 9 shown the temporal changes for one of the fields. Field size are much more congruence than BB size due to substance dose fall‐off out of the field.

**Fig. 10:**
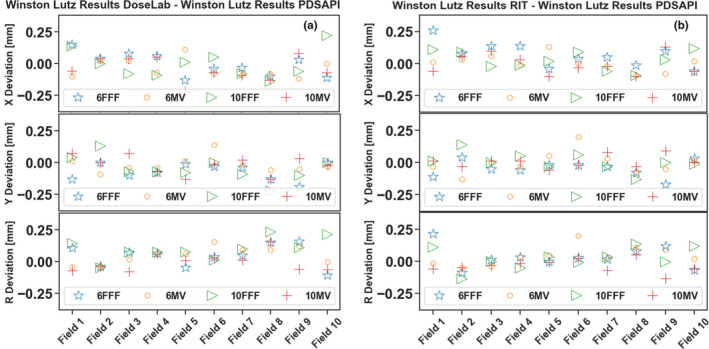
PDSAPI accuracy at different energies. (a) PDSAPI vs DoseLab, (b) PDSAPI vs RIT.

For all the energies we investigated, no substantial energy dependence was observed on PDSAPI accuracy (Fig. 10). All of the differences from RIT or DoseLab are within 1 pixel size.

**Fig. 11:**
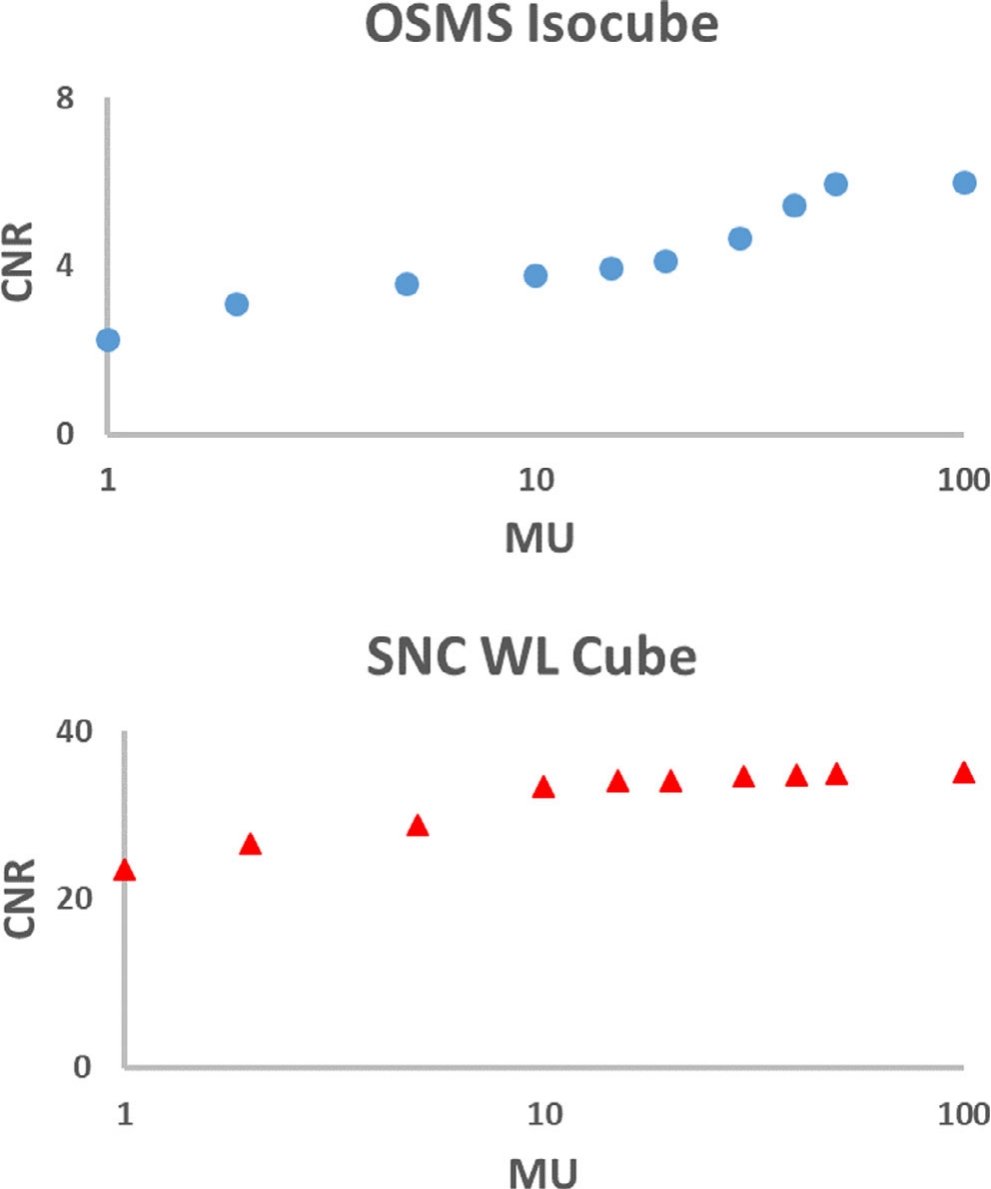
CNR of both cubes varies with MUs.

As shown in Fig. 11, one MU resulted in CNR as high as 23 for SNC cube, while at least 30 MU is needed to have CNR close to 5 for OSMS Isocube.

## 4 Discussion

For 1D comparison against PDSAPI using a 1/3 pixel width threshold, RIT comparison has the minimal pass rate (63.0%), and histogram method is the highest (83.8%). Direct distance measurement method comparison has the highest pass rate (99.5%) for two‐thirds threshold, and comparisons for rest of the methods are similarly in agreement. When using a single‐pixel distance threshold, direct distance measurement method is still the closest one to PDSAPI (100%), while other methods are all equal or above 99.5%. Considering the resolution of portal imager, 1 pixel might be a reasonable critical value for the evaluation. PDSAPI can be a good substitute method for WL test analysis.

**Fig. 12:**
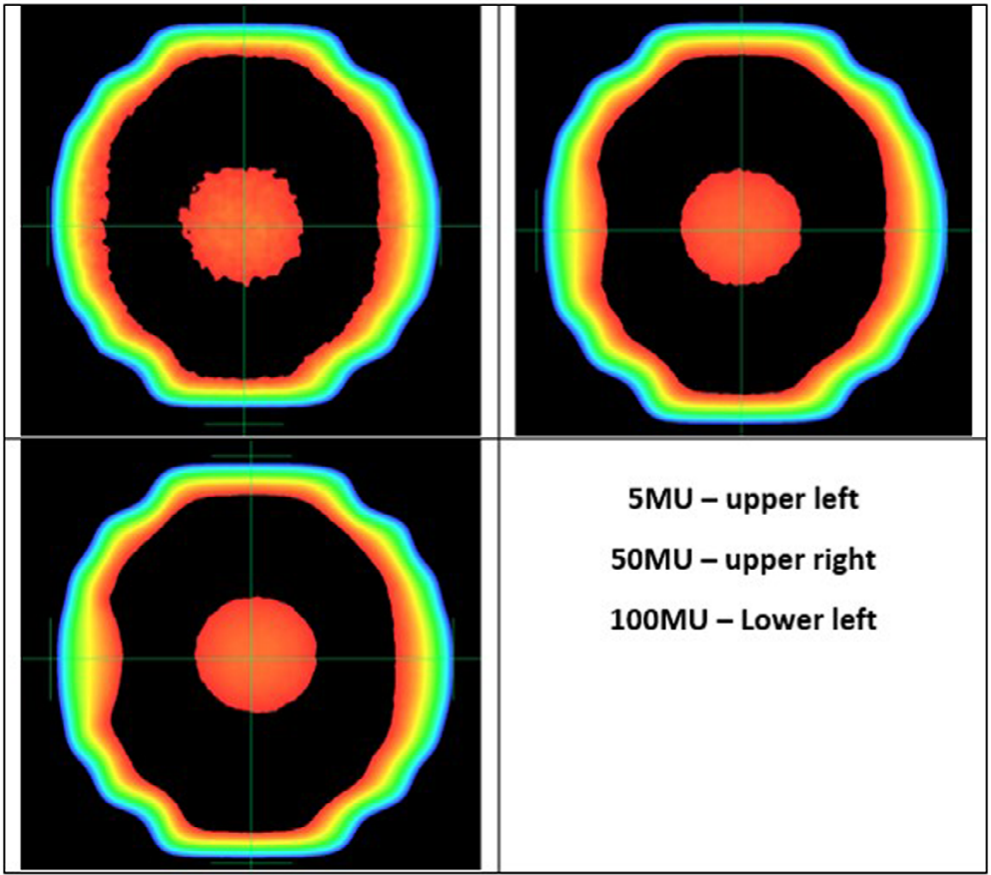
Portal image quality varies with MU.

Several different MU levels were investigated in this study. It is worth mentioning that the factor of how much MU to use during this test is important. As shown in Fig. 12, when using 5 MU, it is difficult to delineate the ball boundary within the image. Using 50 MU, the ball becomes more detectable. With 100 MU, most of the statistical noise in pixel values have been washed out. For validation of PDSAPI method, 100 MU was used to reduce the noise level. As shown in Fig. 11, very small MU is sufficient for WL test when metal BB is used. However, the use of the OSMS cube also allows the user to check the OSMS Isocube at the same time. It will be suggested that WL plan be delivered at least 30 MU per field for OSMS Isocube in order to reduce the impact of statistical deviations of phantom measurements. To speed up the delivery, FFF beam with high dose rate is recommended.

**Fig. 13:**
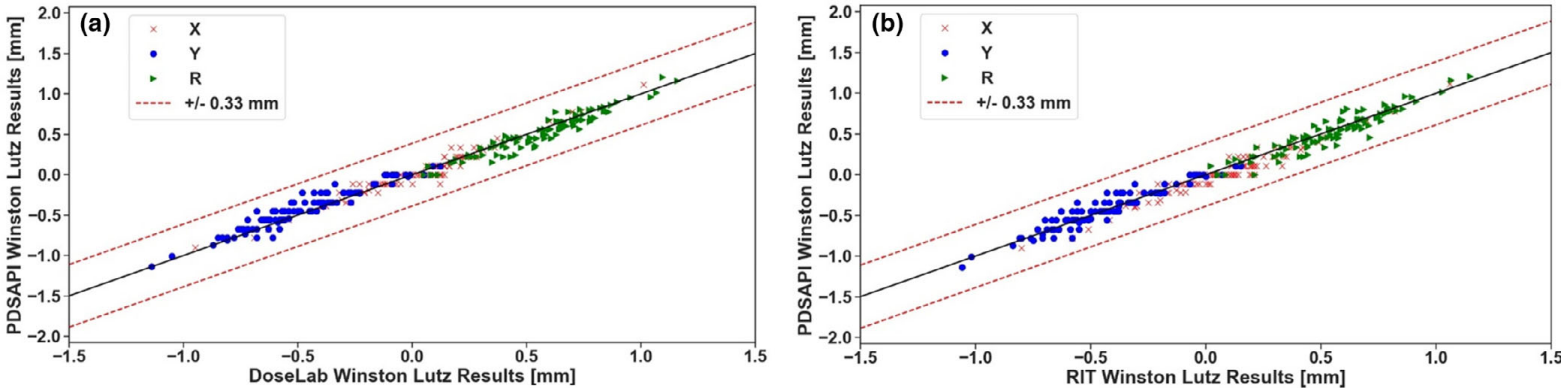
PDSAPI correlation with DoseLab and RIT of SNC Winston‐Lutz cube. (a) Correlation of DoseLab and PDSAPI, (b) Correlation of RIT and PDSAPI.

PDSAPI analysis can be affected by phantom dimension and BB material. In this study, we also investigated SNC WL cube (Sun Nuclear, Melbourne, FL). The cube size is 6 cm × 6 cm × 6 cm with embedded 7 mm tungsten ball at the center. PDSAPI results are within 0.33 mm difference from all other four methods. Similar efficiency of the PDSAPI was observed for 0.22 mm threshold. Substantial improvement was observed for 0.11 mm threshold when PDSAPI comparing against RIT (80.5%) and DoseLab (86.5%) (results are shown in Fig. 13a,b). Comparison against two manual methods is similar as OSMS cube phantom for 2/3 pixel width threshold.

## 5 CONCLUSION

In this study, we developed a PDSAPI to expedite the WL test and to provide documentation capability. This application was tested during our routine clinical WL tests, and the result agrees well with two commercial software as well as two manual measurement methods.

## CONFLICT OF INTEREST

There is no relevant conflict of interest to disclose.
